# 2D Au-Coated Resonant MEMS Scanner for NIR Fluorescence Intraoperative Confocal Microscope

**DOI:** 10.3390/mi10050295

**Published:** 2019-04-30

**Authors:** Cheng-You Yao, Bo Li, Zhen Qiu

**Affiliations:** 1 Department of Biomedical Engineering, Michigan State University, East Lansing, MI 48823, USA; yaochen5@msu.edu; 2 Institute for Quantitative Health Science and Engineering, Michigan State University, East Lansing, MI 48823, USA; libo2@msu.edu; 3 Department of Electrical and Computer Engineering, Michigan State University, East Lansing, MI 48823, USA

**Keywords:** resonant MEMS scanner, electrostatic, parametric resonance, NIR fluorescence, intraoperative microscope, 2D Lissajous, fluorescence confocal

## Abstract

The electrostatic MEMS scanner plays an important role in the miniaturization of the microscopic imaging system. We have developed a new two-dimensional (2D) parametrically-resonant MEMS scanner with patterned Au coating (>90% reflectivity at an NIR 785-nm wavelength), for a near-infrared (NIR) fluorescence intraoperative confocal microscopic imaging system with a compact form factor. A silicon-on-insulator (SOI)-wafer based dicing-free microfabrication process has been developed for mass-production with high yield. Based on an in-plane comb-drive configuration, the resonant MEMS scanner performs 2D Lissajous pattern scanning with a large mechanical scanning angle (MSA, ±4°) on each axis at low driving voltage (36 V). A large field-of-view (FOV) has been achieved by using a post-objective scanning architecture of the confocal microscope. We have integrated the new MEMS scanner into a custom-made NIR fluorescence intraoperative confocal microscope with an outer diameter of 5.5 mm at its distal-end. Axial scanning has been achieved by using a piezoelectric actuator-based driving mechanism. We have successfully demonstrated ex vivo 2D imaging on human tissue specimens with up to five frames/s. The 2D resonant MEMS scanner can potentially be utilized for many applications, including multiphoton microendoscopy and wide-field endoscopy.

## 1. Introduction

The intraoperative microscope has become an emerging bio-imaging technology for clinical applications, including molecular imaging-guided surgery [1]. Other intraoperative imaging tools have been successfully demonstrated, such as wide-field fluorescence [2], confocal [3,4], optical coherence tomography (OCT) [5,6], multiphoton [7,8], etc. Among these state-of-the-art optical imaging modalities, miniaturized fluorescence confocal microscopy holds the promise for many translational applications [3,9], including both early cancer detection and tumor margin delineation. MEMS technology plays an important role in the instrument miniaturization of the intraoperative confocal microscopes, in which MEMS scanners and actuators perform beam steering and focus tuning [10,11]. Various MEMS-enabled confocal microscopes [12,13,14,15,16] have been previously developed by integrating custom-made micro-scanners based on different working principles, such as electromagnetic [17,18], electro-thermal [19,20,21], electrostatic [22], and thin-film piezoelectric [23,24]. Due to their fast-speed scanning up to large angles in a very small footprint, electrostatic micro-scanners have been widely utilized in MEMS-based microscopes [25,26,27,28,29]. However, to date, only a handful of MEMS-based near-infrared (NIR) (>785 nm) fluorescence intraoperative confocal microscopes have been demonstrated [9]. Most of the existing intraoperative confocal microscopes perform either the reflective-mode imaging or visible-range fluorescence imaging. Although it has been used for clinical trials [3], the commercial Cellvizio™ (Mauna Kea Tech, Paris, France) intraoperative confocal microscope has a limited field-of-view (FOV) (<400 µm) without *Z*-axis scan. It uses fiber bundles combined with micro-optics, while bulky galvanometer scanners are utilized in a pre-objective way. It only operates in the visible range (488–640 nm) because the fiber bundles have a low transmission efficiency in the NIR range. One of the greatest challenges in the compact NIR fluorescence intraoperative confocal microscope is collecting enough signal to achieve an adequate signal-to-noise ratio (SNR) since the fluorescence emission signal is weak during in vivo imaging. Therefore, the reflectance efficiency of MEMS scanners has to be sufficiently high to ensure efficient laser excitation and fluorescence collection. Aluminum coating (on the full wafer) without pattern has been commonly used [8,15]. In addition, to avoid creating a short-circuit between comb-drive fingers, only a very thin layer of aluminum coating (less than 50 nm) can be used. The thin aluminum coating layer’s reflectivity will not be sufficient for weak NIR fluorescence detection from tissue specimens. In the NIR range, the Au coating will provide much better reflectivity (>90%), while the reflectivity efficiency of the aluminum coating is relatively low (80%). Unfortunately, very few Au-coated electrostatic MEMS scanners have been demonstrated or mass-produced for custom-made miniature NIR fluorescence intraoperative confocal microscopes. In this project, based on the parametric resonance working principle [30,31], an electrostatic comb-drive-actuated, gimbal frame-based 2D resonant MEMS scanner has been developed and fully integrated into a newly-developed miniature NIR fluorescence intraoperative confocal microscope (outer diameter (OD) 5.5 mm). To achieve a high reflectivity in the NIR range, the new scanner has been coated with a patterned Au/Ti (Ti: adhesion, low stress) coating layer. Resonant scanners offer large tilting angles with a relatively low driving voltage, compared to the conventional electrostatic MEMS scanners based on staggered or angular vertical comb-drives (SVC or AVC) [8,15,22], which usually require a high driving voltage (>100 V, not safe for humans) for large tilting angles in the DC mode with a raster scanning pattern. By taking advantage of the new resonant scanner in our post-objective scanning-based optics, we are able to realize a large FOV (up to 1000 µm) with very low driving voltage (36 V), which is important for in vivo imaging of humans. Our design was inspired by the seminal work [31] by the team led by Schenk. Unfortunately, the former processes are not suitable for our design. A resonant scanner with a large fill-in factor (on the *X*-axis, “dumb-bell” shape) is required to fit the post-objective scanning-based optics for depth imaging with a large FOV. For example, in the former process, KOH-based wet etching on the backside of the wafer required more supporting materials on both the device and handle silicon layers. In our study, based on a single silicon-on-insulator (SOI) wafer, a simplified dicing-free micromachining process has been developed for a mass-production with a high yield. The backside enhancement structures under the micro-mirror help improve the flatness of the mirror surface. A 2D Lissajous pattern scanning strategy [32,33] has been used by actuating the two axes of the scanner in resonant modes with driving frequencies in a tunable range. We have realized 2D imaging with a frame rate of up to 5 Hz, which is sufficient for clinical applications. In the following sessions, we will describe the 2D resonant MEMS scanner and the miniaturized MEMS-based intraoperative confocal microscope.

## 2. 2D Au-Coated Resonant MEMS Scanner Development

### 2.1. Design of the 2D Au-Coated Resonant MEMS Scanner

**Figure 1:**
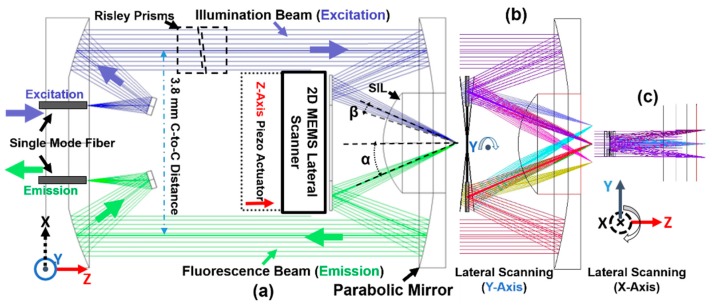
Ray-tracing simulation of the optical design for the MEMS-based intraoperative near-infrared (NIR) confocal microscope’s scan-head (outer diameter (OD): 5.5-mm package). (**a**) Schematic drawing of the scan-head, such as the collimating, focusing, and scanning in the post-objective dual-axis confocal architecture aimed for 3D NIR fluorescence imaging, demonstrating the geometric requirements for the MEMS scanner; a pair of tiny Risley prisms were used for precise alignment; single-mode fibers (S630HP, numerical aperture (NA) = 0.12) were used for delivering and collecting light beams; SIL: solid immersion lens, β: free-space numerical aperture of the individual beams, and α: the intersection half-angle of the beams. (**b**) Lateral scanning around the *Y*-axis of the micro-mirror. (**c**) Lateral scanning around the *X*-axis of the micro-mirror.

The ray-tracing simulation has been studied in the optics design software (ZEMAX, ver. 13, Kirkland, WA, USA) to design the optics and optomechanical system for the scan-head (OD 5.5-mm package) of the miniature fiber-based NIR fluorescence intraoperative confocal microscope, as shown in Figure 1. A 2D MEMS scanner was located at the post-objective position that was close to the distal-end of the parabolic mirror (focus: 4.6, OD 5.0 mm, Al substrate, custom-made by diamond turning). Single-mode fibers (model: S630HP, optimized for the NIR range, NA = 0.12, Nufern, East Granby, CT, USA) have been used for delivering the illumination beam and collecting the fluorescence beam (1/e^2^ diameter ~900 µm); see Figure 1a. More details about the imaging system will be introduced in Section 3.2, including the fiber-based multi-color laser system and the multi-channel fluorescence collection system (mounted on an imaging cart). Although the confocal microscope was designed for multi-color imaging, we only focused on the single-color NIR fluorescence imaging (excitation: 785 nm, emission: >800 nm) in this study. Two collimated and parallel beams (excitation and emission) were weakly focused by the front-side parabolic mirror before being reflected by the MEMS scanner; see Figure 1a. At the center of the parabolic mirror, a solid immersion lens (SIL, fused silica, OD 1.9 mm, full hemisphere, *n* = 1.46, radius: 1.5 mm) contacted the tissue specimens. The distance between the two collimated and parallel beams was 3.8 mm, which were precisely aligned by a pair of Risley prisms (BK7 glass, OD 1 mm, 1 mm thick, wedge angle: 0.1 deg. ±1.5 arcminutes) are shown in Section 3.1. The MEMS scanner steered the light beams with large tilting angles, around the *Y*-axis and the *X*-axis (Figure 1b,c) and achieved a large field-of-view (FOV). Based on a custom-made spring-based mechanism, a *Z*-axis piezoelectrical actuator (P-601.4SL, Physik Instrumente, Karlsruhe, Germany) performed the axial scanning with either a DC stacking mode or a slow scanning mode (<5 Hz); details in Section 3.1. An FOV of 800 µm on the lateral axis can be achieved by scanning the micro-mirror with a ±3° mechanical scanning angle (MSA).

We have used both the ray-tracing simulation and theoretical equations to design and optimize the optics. Both lateral and axial resolutions (full width at half maximum (FWHM), theoretical) may also be calculated using the following Equations (1) and (2), assuming Gaussian beams:

(1) $$\Delta \mathrm{Res}\_\mathrm{lateral}=\frac{0.466\lambda }{n\beta \cos\left(\alpha \right)},$$

(2) $$\Delta \mathrm{Res}\_\mathrm{axial}=\frac{0.466\lambda }{n\beta \sin\left(\alpha \right)},$$

where ∆Res_lateral is the lateral resolution, ∆Res_axial is the axial resolution, *n* is the index of refraction (assuming *n* = 1.4), *β* is the free-space numerical aperture (NA) of the individual beams (excitation and emission), and *α* is the intersection half-angle of the beams. *β* and *α* have been illustrated in Figure 1a. *λ* = 0.785 µm (NIR light), *β* = 0.128, *θ* = 0.419 rad (24°). We have calculated the theoretical resolutions of the optics: ∆Res_lateral = 2.24 µm, ∆Res_axial = 5.02 µm. Based on the theoretical values, the confocal microscope may potentially provide cellular-resolution imaging.

**Figure 2:**
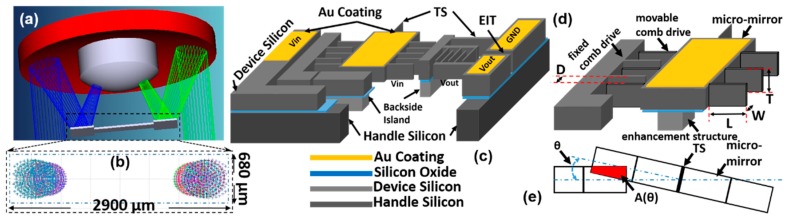
Schematic drawing of the 2D patterned Au-coated resonant micro-scanner and its electrical layout. (**a**) CAD drawing of the scanning micro-mirror inside the confocal microscope’s scan-head (OD: 5.0 mm). (**b**) Zoom-in view of the ray-tracing beam spots (changes due to axial scanning, 0–400 µm), effective area of the “dumbbell” shaped micro-mirror. (**c**) Schematic drawing of the device silicon/buried oxide/handle silicon layers (not to scale), in-plane comb-drive actuator fingers, Au coating, inner and outer torsion springs, electrical insulation trenches, gimbal frame, backside island, and electrical layout, not to scale Note: EIT, electrical insulation trench; TS, torsion spring. (**d**) Schematic drawing of the fixed and movable comb-drive actuator fingers, D: the distance (or gap) between the comb-drive actuator fingers, W: width, L: length. (**e**) Schematic drawing of the actuation using the in-plane comb-drive actuator fingers, *θ*: tilting angle, A(*θ*): overlapped area.

For clinical applications, miniaturized intraoperative NIR confocal microscopies usually require high sensitivities for the emitted weak fluorescence signals, a large FOV with a low driving voltage (safety voltage 36 V), and compact form factors. Therefore, a parametrically-resonant 2D MEMS scanner with a patterned Au-coated surface (>90% reflectivity for an NIR > 785-nm wavelength) will be an ideal choice for the scan engine inside the confocal microscopes. In this project, the proposed gimbal frame-based 2D MEMS scanner was an advanced design based on our previous 1D parametrically-resonant scanner design [34,35] for miniaturized confocal microscopes [29]. As shown in Figure 2a,b, the geometric requirements for the 2D MEMS scanner have been determined in the ray-tracing simulation and the CAD drawing of the distal end of the fiber-based confocal microscope’s scan-head (OD 5.5 mm). The actual beam width (1/e^2^) on the micro-mirror changed (Figure 2b) due to the axial scanning along the *Z*-axis from 0–400 µm. An effective “dumbbell shaped” reflective area of 680 by 2900 µm^2^ (Figure 2b) will be sufficient for steering the excitation and emission beams. The 2D MEMS scanner will be micro-machined using a single SOI wafer (40 µm device silicon/2 µm buried oxide/500 µm handle silicon). It used an in-plane comb-drive actuator configuration, as shown in Figure 2c–e, based on the parametric resonance working principle.

For each axis of the 2D parametrically-resonant MEMS scanner, the equation of dynamic motion is essentially governed by the theory from the seminal work on parametric resonance [30]:

(3) $$J\ddot{\theta }+c\dot{\theta }+k\theta =F\left(t,\theta \right)$$

where *θ* is the tilting angle, as shown in Figure 2e, *J* is the mass moment of the inertia, *k* is the torsion spring stiffness constant, *c* is the average damping constant, and *F* is the applied torque. The parameters were used for either the outer gimbal frame or the inner micro-mirror.

The applied torque *F* and the capacitance *C* are defined as the following Equations (4) and (5):

(4) $$F=N\frac{1}{2}\frac{dC}{d\theta }V^{2}\left(t\right),$$

(5) $$C=\frac{\varepsilon_{0}\varepsilon_{r}A\left(\theta \right)}{D},$$

where *N* is the number of comb-drive fingers on one actuation side, *C* is the capacitance between comb-drive fingers, *dC*/*dθ* is the rate of change of the capacitance for one comb-drive finger with respect to the angular displacement, *V*(*t*) is the driving signal (a periodic square waveform with a 50% duty cycle was used in our study), *ε*_0_ is the electric constant (8.8542 × 10^−12^ F m^−1^), *ε*_r_ is the relative static permittivity (1.0 for the ambient air), *A*(*θ*) is the overlapped area of the electrodes and two comb-drive actuator fingers, and *D* is the distance (or gap) between the electrodes and two comb-drive fingers.

Equations (3)–(5) have been used to guide the 2D resonant MEMS scanner design for both the gimbal frame (outer axis) and the micro-mirror (inner axis), assuming there is no cross-talk between these two axes. According to Equations (4) and (5), to maintain large applied torques *F*, low driving voltages can be achieved by increasing the number (*N*) and the capacitance (*C*) of the comb-drive fingers. While we designed the torsional springs for both axes, the tilting (or torsional) modes have to be the dominant vibration modes, and the modal analysis in ANSYS has confirmed that the eigenfrequencies (natural frequencies) of other modes were well separated from the basic tilting (torsional) mode frequencies.

**Figure 3:**
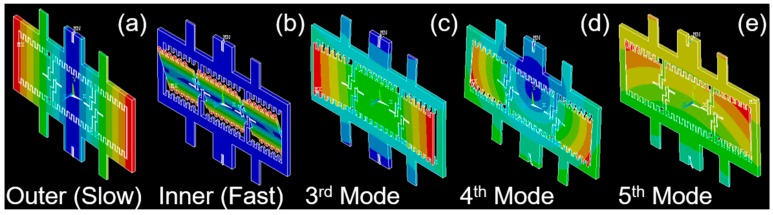
Finite element analysis (FEA) simulation and modal analysis in ANSYS for the 2D parametrically-resonant MEMS scanner. (**a**) Outer (slow) tilting around the *Y*-axis (resonant frequency: ~1090 Hz); (**b**) inner (fast) tilting around the *X*-axis (resonant frequency: ~6250 Hz); (**c**–**e**) higher order resonant modes are designed to be far away from the basic tilting modes around the X- and Y-axes, 3rd~5th mode: ~11,230 Hz, ~13,830 Hz, ~13,870 Hz, respectively.

In Table 1, we have listed the details about the structures. These features have been chosen mainly based on the three factors: (1) to meet the requirements in the system-level optics design and electrical layouts; (2) to consider the realistic capabilities of the microfabrication tools; (3) empirical experience. For example, the gimbal frame-based 2D resonant MEMS scanner design used electrical insulation trenches (EIT, 5 µm gap) for dividing the electrical layouts on the inner and outer axes. The width (W) and the gap (D) for the individual comb-drive fingers were designed to be 5 µm based on the capabilities (like aspect ratio) of the deep reactive ion etching (DRIE) tool. For the outer gimbal frame, four banks of comb-drive fingers have been designed on each side (symmetric on the chip); see Figure 3. In each bank, there were *N* = 22 comb-drive fingers. For the inner micro-mirror, there were *N* = 115 comb-drive fingers on each side (symmetric). The length (L), gap (D), and number (*N*) of comb-drive fingers have been chosen to ensure sufficiently large driving torque at a low driving voltage (~36 V) and to avoid the lateral pull-in effects. The comb-drive fingers, the torsion springs, the gimbal frame, and the scanning micro-mirror were all on the device silicon layer (40 µm thick) of the SOI wafer. Without filling in the trenches with silicon dioxide or silicon nitride [31], the gimbal frame was held by a backside island layer (around 50 µm thick) formed by the backside step-etching process on the handle silicon layer (500 µm thick) during the microfabrication process. As shown in Figure 3, inside the gimbal frame, on the four-bar inner piers, eight evenly-distributed inner torsional springs minimized the interruption of the light beam on the effective area and potentially reduced the overall stress during dynamic scanning. By taking advantage of the backside step-etching process (the same process for the backside island under the gimbal frame), the enhancement structures (~50 µm thick) will also be micro-machined under the scanning micro-mirror; see Figure 2d. The overall optical quality of the micro-mirror benefited significantly from both enhancement structures and the high-quality patterned Au/Ti coating (Ti: adhesion layer). Instead of using Cr, the Ti material has been chosen because it had less residual stress after deposition. In the previous research on various MEMS scanners, full-wafer non-patterned metallic (Al or Au) coating approaches have been commonly used [8,15]. In those processes, only a thin metallic layer (less than 500 Å) can be coated to avoid creating a short-circuit (especially on the comb-drive fingers). An alternative way for the patterned metallic coating is to use a shadow mask at the end of the microfabrication process flow. However, the shadow mask-based coating process could still potentially shorten the comb-drive fingers or encounter serious misalignment issues due to the relatively coarse alignment (compared to precise lithography). Our proposed patterned (by lithography) Au/Ti coating, at the beginning of the microfabrication process, led to a superior optical quality for the NIR fluorescence microscopy applications, because of two main factors: (1) accuracy of the reflective area (other non-effective areas will not reflect light); and (2) relatively thick (>1000 Å) metallic coating for a high reflection coefficient (>90%).

By using the modal analysis in ANSYS, the 2D parametrically-resonant MEMS scanner’s eigenfrequency (natural frequencies) analysis has been studied; see Figure 3. Based on the parametric resonance working principle [30], the driving frequency was twice the resonant frequency over n (*f*_driving_ = 2 × *f*_resonant_/*n*, *n* = 1, 2, 3, …, N). In ambient air at room temperature, the resonant mode can be observed with N ranging from 1–4, depending on the design of gimbal structures and torsion springs. From the modal analysis, the outer gimbal frame’s tilting mode resonant frequency was around 1090 Hz (slow, around *Y*-axis); see Figure 3a. The inner micro-mirror’s tilting mode resonant frequency was around 6250 Hz (fast, around the *X*-axis); see Figure 3b. The combination of the inner and outer resonant frequencies was carefully designed for the en face 2D imaging with a 2D Lissajous scan pattern (up to five frames per s). Other higher order resonant modes have also been designed (third mode: ~11,230 Hz, fourth mode: ~13,830 Hz; fifth mode: ~13,870 Hz) to ensure that they were far away from the first two basic tilting (torsional) modes.

### 2.2. Microfabrication of the 2D Au-Coated Resonant MEMS Scanner

**Figure 4:**
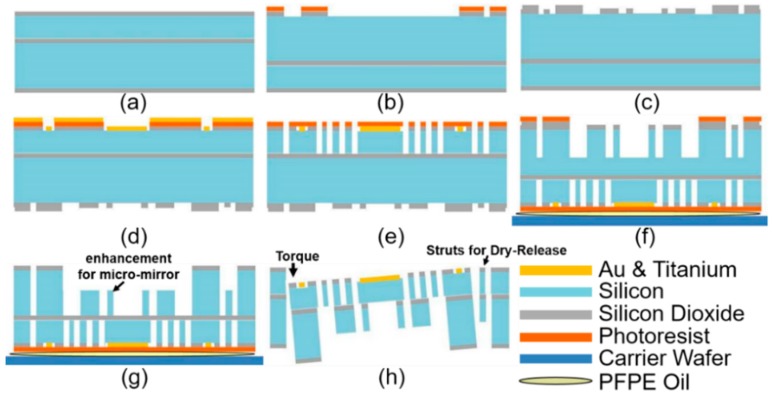
Dicing-free single SOI-wafer-based microfabrication process flow for the 2D patterned Au-coated resonant MEMS scanner with backside enhancement structures. (**a**) Wafer cleaning and the SiO_2_ hard mask layer preparation on both sides of the SOI wafer using LPCVD; (**b**) patterning the backside SiO_2_ hard mask for the scanner’s full chip frame on the handle silicon layer (Mask 1, handle silicon chip frame); (**c**) patterning the backside SiO_2_-based hard mask (Mask 2, island); this SiO_2_ layer was prepared by PECVD; (**d**) lift-off process for a patterned Au/Ti coating (1000 Å/50 Å) to form the reflective surface for the NIR light and the alignment marks on the scanner (Mask 3, Au coating); (**e**) front-side DRIE process on the device silicon layer (Mask 4, device silicon); (**f**) DRIE process on the backside handle silicon layer; the PFPE oil was used for enhancing the thermal conductivity between the SOI and the carrier wafer; (**g**) DRIE process on the backside handle layer till reaching the buried oxide layer; (**h**) wet etching of the SiO_2_ under the scanning micro-mirror using buffered hydrofluoric acid (buffered oxide etch (BOE) 7:1); the torque was applied using a sharp tweezer tip to break the struts so that the individual MEMS chip (3.2 by 2.9 mm^2^) would be dry-released and harvested with a high yield (over 80%).

**Figure 5:**
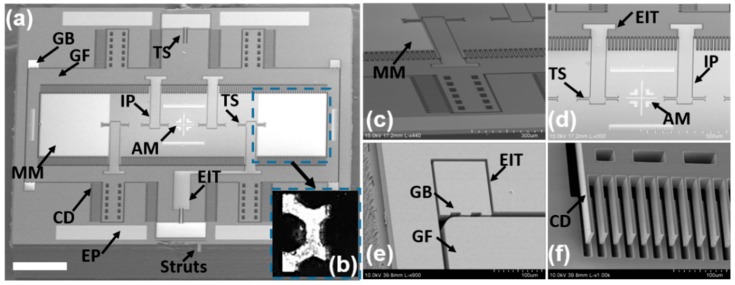
SEM and stereomicroscopic images of the 2D Au-coated resonant MEMS scanner. (**a**) MEMS chip with a 3.2 mm (horizontal) × 2.9 mm (vertical) footprint, scale bar: 500 µm; (**b**) zoom-in view stereomicroscopic image of the backside enhancement structures (50 µm thick) under the Au-coated scanning micro-mirror; the image was taken when the chip was upside down; (**c**) gimbal frame with the inner micro-mirror; (**d**) inner micro-mirror with “cross” shaped alignment marks at the center and two paralleled rulers; (**e**) edge of the gimbal frame with its protection bumper for the sidewall stop to avoid shorting the electricity; (**f**) comb-drive actuator fingers on the outer gimbal frame; notes: the gimbal frame was tilted with pre-loaded torque to expose the sidewall of comb-drive actuator fingers during SEM. Notes: GB, gimbal frame’s bumper; GF, gimbal frame; AM, alignment marks; MM, micro-mirror coated with Au/Ti; TS, torsion spring; IP, inner piers for micro-mirror; EP, electrical pads; CD, comb-drive actuator fingers.

Based on a four-inch SOI wafer, a four-mask, dicing-free microfabrication process has been developed for a high-yielding (>80%) mass-production of the 2D resonant MEMS scanners. The SOI wafer consisted of a device silicon layer (40 µm thick), buried oxide layer (BOX, 2 µm thick), and a handle silicon layer (500 µm thick). As shown in Figure 4, MEMS chips (footprint size: 3.2 by 2.9 mm^2^) were dry-etched and dry-released through three primary etching steps using the DRIE process (SPTS Pegasus, fluorine-based Bosch Process; etching rate was ~6 µm/min). The process started with a high-quality silicon dioxide (SiO_2_, 2 µm thick) layer deposition on both sides of the SOI wafer using the low pressure chemical vapor deposition (LPCVD) process equipment: the Tempress system for low temperature oxide (LTO)); see Figure 4a. The standard oxidation recipe for the LPCVD process is shown as follows: 425 °C temperature, 150 mTorr pressure, O_2_ gas flow rate of 225 standard cubic centimeters per minutes (SCCM), N_2_ gas flow rate of 100 SCCM, SiH_4_ gas flow rate of 75 SCCM. Then, the backside the SiO_2_ layer was patterned (Mask 1, handle Si chip frame) with the reactive ion etching (RIE) process (LAM 9400); see Figure 4b. The key parameters in the recipe of the SiO_2_ etching process are listed as follows: 2.3 mTorr pressure, CHF_3_ gas flow rate of 5 SCCM, bias voltage 250 V. A thin photoresist layer (SPR 220, 5 µm thick, 2500-rpm spin speed, 30-s soft-bake, step-down to 115 °C/90 s MicroChem, Westborough, MA, USA) has been used for the patterning of SiO_2_. The SiO_2_ layer essentially performed as a hard mask for the handle silicon layer’s full-depth etching in Figure 4g. As shown in Figure 4c, another SiO_2_ layer (2 µm thick) by the plasma-enhanced chemical vapor deposition (PECVD) process (GSI ULTRADEP 2000, GSI Lumonics, Novanta Inc., Bedford, MA, USA) on the handle silicon layer was patterned (Mask 2, island) for the step-etching process, to form the islands under the gimbal frame and the enhancement structures under the micro-mirror. For the SiO_2_ deposition by the PECVD process, the key parameters of the deposition recipe are listed as follows: temperature 250 °C, SiH_4_ gas flow rate of 100 SCCM, N_2_O gas flow rate of 300 SCCM, RF power 22 W. The Au/Ti (thickness: 1000 Å/50 Å; Ti: adhesion) metallic coating layer was first prepared by an evaporation machine (Enerjet) onto the front-side device silicon layer. As shown in Figure 4d, the Au/Ti metallic coating layer was then patterned (Mask 3, Au coating) by the lift-off process to form the reflective surface on the scanning micro-mirror for the NIR light beam, electrical pads, and the alignment marks around the chip. The metallic coating layer will be fully protected through the rest of the processes so that the effects of the sequential processes on the surface roughness are minimal. The structures with fine features (up to 5 µm resolution) on the front-side device silicon layer (40 µm thick) were formed by the DRIE process (Mask 4, device Si). These important structures include a scanning micro-mirror, a gimbal frame, comb-drive actuator fingers (100-µm length, 5-µm width, 5-µm gap), outer torsion springs (two springs, each had a 10-µm width, 175-µm length), inner torsion springs (eight, each had a 5-µm width, 100-µm length), and electrical insulation trenches (5-µm gap); SEM images are shown in Figure 5. During the DRIE process, an inductively-coupled plasma (ICP) source has been used (825 W power, 2 M Hz). In the chamber, several key parameters have been controlled: 23 mTorr pressure, 40 °C coil temperature, 20 °C substrate temperature, SF_6_/Ar (100/40) with bias (9 W). Before processing the backside of the SOI wafer, the front-side features had to be fully protected by spin-coating thick photoresist (AZ9260, 10 µm thick, spin speed 2000 rpm, soft-bake 110 °C/180 s, Clariant Corporation, Muttenz, Switzerland), which not only covered the surface, but also filled in the trenches on the device silicon. A thick photoresist layer (AZ9260, 8 µm thick, spin speed 3000 rpm) needed to be patterned one more time (Mask 1, handle silicon chip frame) on the backside handle Si layer; see Figure 4f. During the whole backside step-etching process (Figure 4f,g), the four-inch wafer needed to be fully attached to the six-inch carrier wafer with thermally-conductive perfluoropolyether (PFPE) oil, which would significantly enhance the thermal transfer. The wafer or photoresist burning incidents are common issues in the development of MEMS scanners. These problems usually occur due to overheating or poor thermal transfer during the dry etching of the thick handle silicon layer (500 µm) at the full wafer level, especially when the front-side device silicon layer is already full of structures, such as the trenches and comb-drive fingers. The PFPE oil helped resolve the thermal transfer problems by enhancing the contact between the carrier wafer and the SOI wafer. The step-etching process on the handle silicon layer had to be precisely time controlled by reaching the buried oxide layer; see Figure 4g. The step-etching process on the handle silicon layer would form two critical structures (around 50 µm thick): (1) the backside island for supporting the outer gimbal frame of the MEMS scanner; and (2) the enhancement structure under the micro-mirror for a high optical quality. Finally, the buried oxide layer under the moving structures on the device silicon layer (such as the scanning micro-mirror, torsion springs, and the gimbal frame) was released by a buffered oxide etch (BOE, 7:1); see Figure 4h. The conventional dicing saw-based “wet” cutting process at the last step of the microfabrication process could potentially damage the fragile micro-mirrors or gimbal frames that were linked to the substrates only by a few torsion springs. These issues can be avoided in the newly-developed dicing-free process by combining front-side and backside DRIE, leading to improved die yielding (>80%) and MEMS chips with arbitrary contours. The individual chips were dry-released from the SOI wafer by manually breaking the struts (link arms, 15 µm width) on the edge of the chip, in Figure 4h and Figure 5a, with either the laser cutting dry process [35] or the torque applied by a sharp tweezer tip. A custom-made MEMS probe station (Model S-725PLM&-PRM, Signatone, Gilroy, CA, USA) has been used for screening the dry-released chips, which would be integrated into the distal end scan-head of the miniaturized intraoperative NIR fluorescence confocal microscope.

Scanning electron micrograph (SEM) and stereomicroscopic images of the micro-machined device are shown in Figure 5. The backside enhancement structures under the micro-mirror (Figure 5b) have improved the flatness of the micro-mirror and compensated for the residual stress induced by the Au/Ti coating (note: the image was taken when the chip was upside down). The struts (link arms, 15 µm) on the edge of the MEMS scanner were designed for the dry-releasing from the SOI wafer and can be easily broken manually by the sharp tip of a tweezer, and the residual arms are shown in Figure 5a. Different structures with fine features are shown in Figure 5c–f, including the micro-mirror, torsion springs, electrical insulation trenches, alignment marks, electrical pads, bumpers, the gimbal frame, and comb-drive fingers. The electrical pads, the coating on the micro-mirror, and the alignment marks were all formed at the same time by the Au/Ti coating and the lift-off process; see Figure 4d.

### 2.3. Characterization of the 2D Au-Coated Resonant MEMS Scanner

On the scanning micro-mirror surface, the patterned and relatively thick Au/Ti coating (1000 Å/50 Å) with a low residual stress guaranteed the high reflectivity (>90%) in the near infrared (NIR) range with low scattering losses [24,35]. The confocal microscopy (LEXT, Olympus, Tokyo, Japan) characterized the surface of the micro-mirror (at the static status) after microfabrication. Due to the backside enhancement structures, the micro-mirror of the MEMS scanner had a radius of more than 1800 mm with a peak-to-valley surface deformation <0.1 μm. The large radius (>1.8 m) of the micro-mirror’s curvature proved that the stress of the micro-mirror was relatively low. Otherwise, the micro-mirror would either bend (small curvature radius) or even twist, which would induce serious misalignment or non-focus problems in the optics. These measurement results showed that the micro-mirror had a surface roughness of <5 nm. These characteristics will ensure high-quality imaging.

**Figure 6:**
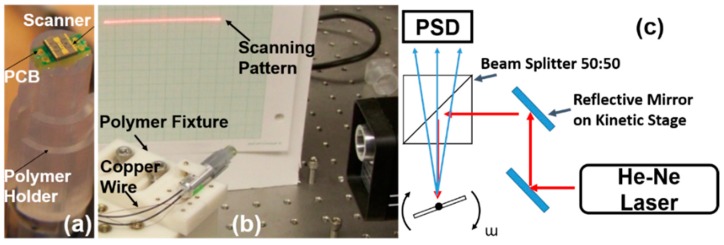
Characterization of the 2D Au-coated resonant MEMS scanner using a position sensing detector (PSD)-based measurement system setup. (**a**) Simple packaging of the MEMS device onto the PCB stage mounted on a 3D-printed polymer holder; (**b**) scanning pattern demonstration by clamping the holder on the 3D-printed polymer fixture; thin protected copper wires were used for delivering electricity to the MEMS scanner; (**c**) schematic drawing of the custom-made measurement system setup for fully characterizing the 2D resonant MEMS scanner; the PSD was located behind the 50:50 beam splitter; two silver-coated reflective mirrors held on kinetic stages were used to align the He-Ne laser beam accurately onto the surface of the scanning micro-mirror; the steered laser beam passed through the beam splitter and was detected by the PSD with fast responses.

**Figure 7:**
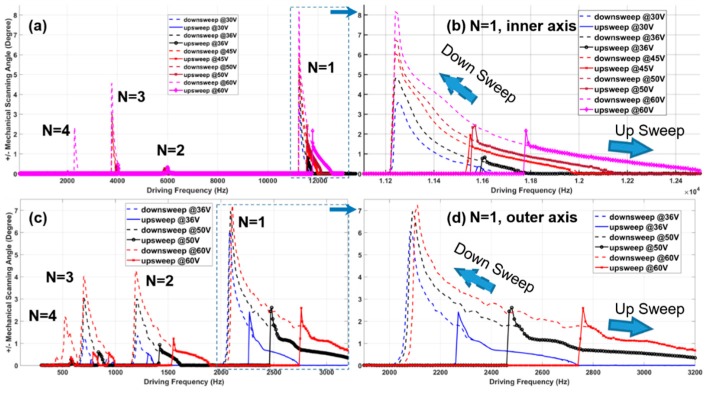
Characterization of the parametrically-resonant scanner on both inner (fast) and outer (slow) axes. (**a**) Tilting amplitude (mechanical scanning angle (MSA), units: degrees) vs. driving frequency sweeping (from 1 Hz–13k Hz, in ambient air, with variant driving voltage from 30 V–60 V) of the inner (fast) axis (N = 1–4); (**b**) zoom-in view of the response curves of the inner (fast) axis while N = 1; (**c**) tilting amplitude vs. driving frequency sweeping (from 1 Hz–3500 Hz) of the outer (slow) axis (N = 1–4, in the ambient air at room temperature, with variant driving voltage from 36 V–60 V); (**d**) zoom-in view of the response curves of the outer axis for N = 1. Note: while N = 1, the driving frequency is double the resonant scan frequency.

To demonstrate the scanning performance, the MEMS scanner was bonded onto a printed circuit board (PCB) mounted on a custom-made polymer holder with an outer diameter of 10 mm; see Figure 6a,b. The polymer holder was clamped onto a polymer-based fixture, including a V-groove and a cube. Most of the polymer-based fixtures were custom-made by a 3D printing tool (SLA, 3D Systems). Using a He-Ne laser, the scanning pattern can be observed directly on the paper screen; see Figure 6b. In addition, the dynamic characteristics and the parametrically resonant frequency responses have been characterized by using a He-Ne laser-based steering beam measurement setup; see Figure 6c. The laser beam from the He-Ne Laser (633-nm wavelength) was normally incident on the scanning mirror surface of the 2D resonant MEMS scanner and then reflected onto a position sensing detector (PSD, OnTrak, Irvine, CA, USA) that was in front of a beam splitter (50:50). A single data acquisition card (DAQ, PCI-6115, National Instrument, Austin, TX, USA) was utilized for sending out analog driving signal outputs (two-channel 12-bit analog outputs, 2.5 mega-samples per second—MS/s dual channel) and acquiring position sensing signals (two out of four-channel high-speed analog inputs, 10 MS/s per channel). The outer and inner axes of the MEMS scanner were driven by a two-channel high-voltage amplifier (gain = 20, Model 2350, Tegam), which was connected to the DAQ card by BNC cables. Figure 7 shows the response curves’ characterization of both the inner and outer axes with various driving voltages. The resonant MEMS scanner was driven by a square waveform of various driving voltages (V peak-to-peak ranged from 30–80 V) with the frequency sweeping (from 1 Hz–13k Hz, up and down sweep) at the pulse width modulation (PWM) duty cycle of 50% (*f*_driving_ = 2 × *f*_resonant_/*n*, *n* = 1, 2, 3, 4, …, N). In the ambient air at room temperature, the parametric resonance phenomena can be easily observed from N = 1–4 with driving voltages lower than 60 V. For the inner (fast) axis, the mechanical scanning angle (MSA) ±4° can be achieved while the driving frequency (*f*_driving_) was around 11.3 k Hz (resonant frequency f_resonant_ = 5659 Hz, *N* = 1, at 36 V). For the outer (slow) axis, the scanner performed tilting with MSA = ±4°, while *f*_driving_ = 2.1 k Hz (*f*_resonant_ = 1050 k Hz, *N* = 1, 36 V). Compared to the simulated results from the modal analysis in ANSYS, the resonant frequency’s real value of both the inner and outer axes was slightly lower due to the manufacturing tolerances and slight over-etching effects on the torsional springs and backside island structures. The phase delay information can be derived by comparing the driving signals (square waveform) from PCI-6115 DAQ (National. Instruments, Austin, TX, USA) with the position sensing signals (sinusoid waveform) acquired by the synchronized channels on the DAQ card. The phase delay values on both inner and outer axes will be critical for the confocal microscopic imaging system, including the open-loop control, the synchronization for data acquisition, and the real-time image reconstruction.

## 3. System Development of the MEMS-Based Near-Infrared (NIR) Intraoperative Confocal Microscope

### 3.1. 2D Resonant MEMS Scanner Integration in the Intraoperative Confocal Microscope

**Figure 8:**
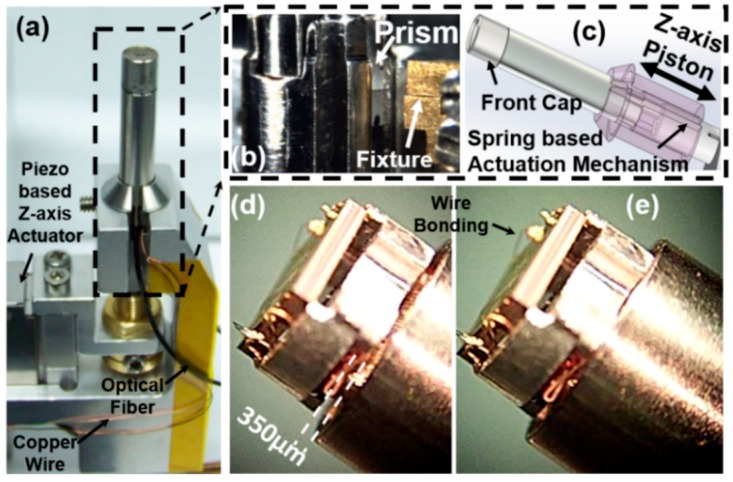
Integration of the 2D resonant MEMS scanner in the miniaturized fiber-based intraoperative NIR fluorescence confocal microscope. (**a**) Stereomicroscopic image of the microscope; a piezoelectrical *Z*-axis actuator (P-601.4SL) was used for axial scanning (DC stacking mode, or axial scanning up to 5 Hz with a 350-µm range); (**b**) zoom-in view of the microscope’s distal end; Risley prisms were used for aligning the beams precisely to be parallel; additional metal alignment fixtures; (**c**) schematic drawing of the scan-head’s distal end and the spring-based *Z*-axis piston mode actuation mechanism; (**d**,**e**) zoom-in view of the *Z*-axis movement of the MEMS scanner holder stage with 350-µm movement; the front cap has been taken off.

The 2D resonant MEMS scanner required precise alignment based on the on-chip alignment marks under the stereomicroscope (SMZ660 ESD Microscope, Nikon, Tokyo, Japan). As shown in Figure 8, the new 2D resonant MEMS scanner has been integrated into the distal scan-head of the miniaturized intraoperative confocal microscope. The MEMS scanner has been wire-bonded manually by a wedge wire bonder (Model 7476, Westbond, Anaheim, CA, USA) using gold wires; see Figure 8e. Additional conductive silver epoxy will be deposited onto the bonding pads to ensure the steady electrical connections aimed for longitudinal studies; see Figure 8d,e. As shown in Figure 8a, the distal end of the fiber-based confocal microscope prototype has an outer diameter of 5.5 mm, three thin protected copper wires delivered electricity to the MEMS scanner (*V*_in_, *V*_outer_, Ground- GND). The piezoelectrical actuated spring mechanism drove the MEMS scanner holder forward and backward (at the distal end of scan-head, dashed line), in Figure 8a,c. Two tiny Risley prisms (OD 1.0 mm) were used to align the dual beams precisely to be parallel; see Figure 8b. Additional metal fixtures helped the alignment and will be disassembled while the system integration is fully accomplished; see Figure 8b. The *Z*-axis piezoelectrical actuator (P-601.4SL, Physik Instrumente, Karlsruhe, Germany) was used for the axial scanning (DC stacking mode and slow *Z*-axis scanning with 5 Hz or less); see Figure 8c. Figure 8d,e essentially demonstrates the axial scanning of the MEMS scanner holder driven by the *Z*-axis piezoelectrical actuator (P-601.4SL) through the spring mechanism (350-µm range, up to 5 Hz, sufficient for vertical cross-sectional imaging with a raster scanning pattern).

### 3.2. Fiber-Based Fluorescence Imaging System Development

A single DAQ card (PCI-6115, deep onboard memory = 32 MS) has been utilized for the NIR fluorescence intraoperative confocal microscopic imaging system. A custom-made NI-DAQmx-based LabVIEW (National Instrument) program has been developed for controlling the 2D resonant MEMS scanner and the piezoelectrical actuator (P-601.4SL) with feedback controls, acquiring fluorescence signals, and reconstructing 2D images.

**Figure 9:**
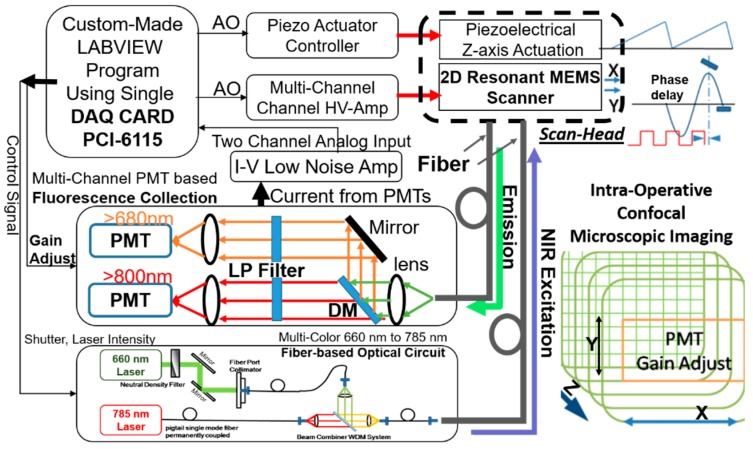
Schematic drawing of the NIR fluorescence confocal imaging system, including the fiber-based multi-color laser excitation and multi-channel fluorescence emission collection system based on photomultiplier tubes (PMT), control system, signal amplification, data acquisition, and real-time image reconstruction; the gain settings of the PMTs were adjusted along the *Z*-axis for depth imaging. Note: LP filter: long-pass filter, DM: dichroic mirror.

As shown in Figure 9, a multi-color fiber-coupled laser engine has been developed for the intraoperative confocal microscope system (excitation with 660-nm and 785-nm CW lasers from Toptica company, Munich, Germany; more lasers can be added inside; only a 785-nm CW laser was used in this study). The fiber-based multi-channel fluorescence collection system has been developed for splitting the fluorescence emission signals acquired from the confocal microscope, in Figure 9. The fluorescence emission light through the single-mode fiber (FC/PC connector) would be first collimated by an aspheric lens (C240TME-B, Thorlabs, Newton, NJ, USA) and then collected by the photomultiplier tubes (PMT, H7422P-50, Hamamatsu, Japan) through a dichroic mirror (cut-off wavelength: 750 nm), long-pass filters, and condense lenses (C240TME-B, Thorlabs). The dichroic mirror split the fluorescence emission lights into two different channels (<750-nm light was reflected, >750-nm passed through). In this study, only the NIR (>800 nm, excited by a 785-nm laser) fluorescence collection channel was used. I-V low noise amplifiers (FEMTO, Model DHPCA-100, no bias, V/A = 10^5^, low-pass 10M Hz, DC coupling) band-pass filter and converted the weak current signal outputs from the PMT detectors, which detected the emitted fluorescence light from the confocal microscope. On the DAQ card, high-speed analog input (10 MS/s per channel) channels digitized the analog signals from the low-noise I-V amplifier, while analog output channels generated the control signal of the 2D MEMS resonant scanner at 1 M samples/s. For fluorescence 2D imaging (up to five frames/s, 2D frame rate), amplified square waveforms (driving voltage Vpp = 36 V) at 11.3 k Hz and 2.1 k Hz drove the inner axis and the outer axis, respectively. Both the X- and Y-axes of the micro-mirror operated in open-loop resonant scanning modes. A 2D Lissajous scan pattern [32,36] has been carefully designed with an optimized frequency combination (validated in a custom-made MATLAB program, but will not be discussed here). For en face 2D imaging on the XY-plane, in the custom-made LabVIEW program, control signals and data acquisition have been fully synchronized for real-time reconstruction of the images by remapping the time series signals to a 2D XY-plane image with a pre-calculated look-up table (LUT) for the 2D Lissajous scan pattern.

### 3.3. NIR Fluorescence Imaging Results of the Intraoperative Confocal Microscope

**Figure 10:**
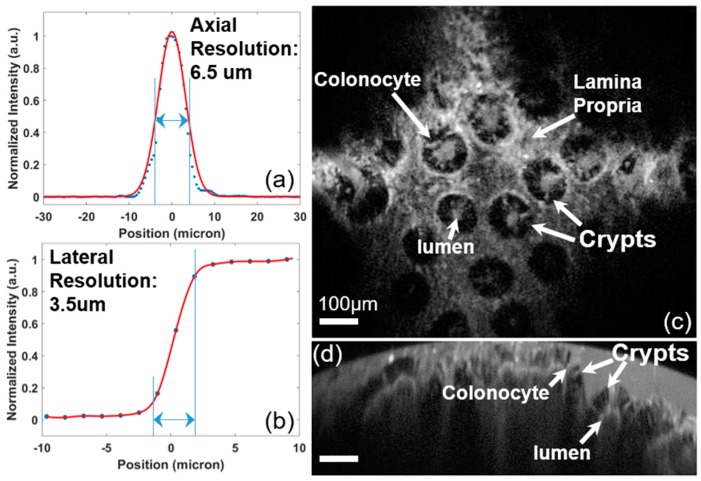
Fluorescence imaging results of the NIR fluorescence intraoperative confocal microscope. (**a**) Lateral and (**b**) axial resolution at the wavelength of 785 nm (focus at 150 µm out of the SIL) by measuring the FWHM in the reflective mode; ex vivo fluorescence images of human colon tissue specimens demonstrated “histology-like” imaging with a large field-of-view (up to 1000 µm), in both the (**c**) en face horizontal cross-sectional image at the 150-µm depth and (**d**) vertical cross-sectional image; crypts, colonocytes, and lumen have been visualized with cellular resolutions, scale bar: 100 µm.

To characterize the NIR fluorescence confocal imaging performance of the 2D resonant MEMS-based intraoperative confocal microscope, lateral and axial resolutions (on-axis) were tested using a knife-edge target at the NIR excitation (785 nm) in the reflective mode. The tested lateral and axial resolutions were 3.5 µm and 6.5 µm, respectively, which were less than the theoretical ones (2.24 µm and 5.02 µm) due to non-perfect optical alignments. Ex vivo fluorescence imaging has also been characterized with human colon tissue specimens that were topically stained with the IRDye800CW dye (excitation: 785 nm), with an approved protocol for human subject research at Stanford University. In this study, we only used an NIR laser (785 nm, 40 mW) and the NIR fluorescence collection channel (>800 nm). The laser power (out of the SIL) into the tissue was ~2 mW. As shown in Figure 10c,d, the MEMS-based intraoperative confocal microscope performed “histology-like” imaging with a large field-of-view (up to 1000 µm). The en face horizontal cross-sectional image (XY-plane) (Figure 10e) was acquired at a *Z*-axis depth = 150 µm in the tissue, while the vertical cross-sectional image shows the XZ-plane with a *Z*-axis depth of 350 µm. In both horizontal and vertical cross-sectional images (Figure 10c,d), crypts, colonocytes, and lumen have been visualized with cellular resolutions. The ex vivo imaging experiments essentially have validated the feasibility of the intraoperative confocal microscope system.

## 4. Conclusions

In this project, we have demonstrated a new 2D patterned Au-coated parametrically-resonant MEMS scanner with a compact form-factor (3.2 by 2.9 mm^2^) for the NIR fluorescence intraoperative confocal microscope system. A dicing-free microfabrication process with a patterned Au coating has been developed for a high-yielding mass-production of the 2D resonant MEMS scanner with an in-plane comb-drive configuration. Both X- and Y-axes of the resonant scanner can achieve large tilting angles ±4° (mechanical scanning angle, ±8° optically) at a relatively low driving voltage (36 V, safe for humans) with a relatively broad tunable driving frequency range. The resonant MEMS scanner has been successfully integrated into a miniaturized NIR fluorescence intraoperative confocal microscope with an outer diameter of 5.5-mm packaging at the distal end. To acquire the NIR fluorescence 2D images, the resonant MEMS scanner operated at the resonant modes on both axes and performed the 2D Lissajous scan pattern. We have demonstrated the ex vivo “histology-like” 2D imaging on human colon tissue specimens with up to five frames/s. The 2D resonant MEMS scanner can also be utilized for other applications, including MEMS-based microendoscopy and wide-field endoscopy.

## Figures and Tables

**Table 1:** Structural features of the 2D resonant MEMS scanner. L, length; W, width; D, distance (gap); T, thickness.

Chip Size (mm)		Comb-Drive Fingers (µm)			Torsion Springs (µm)				Gimbal Frame (mm)		Micro-Mirror (mm)		Backside Island (µm)
					Inner (8)		Outer (2)						
L	W	L	W	D	L	W	L	W	L	W	L	W	T
3.2	2.9	100	5	5	100	5	175	10	3.04	1.36	2.9	0.68	50
